# Bearing Fault Diagnosis Using an Extended Variable Structure Feedback Linearization Observer

**DOI:** 10.3390/s18124359

**Published:** 2018-12-10

**Authors:** Farzin Piltan, Jong-Myon Kim

**Affiliations:** Department of Electrical, Electronics and Computer Engineering, University of Ulsan, Ulsan 680–749, Korea; piltan_f@iranssp.org

**Keywords:** bearing fault detection, feedback linearization observer, model-reference fault diagnosis, variable structure observer, proportional integral observer

## Abstract

The rolling element bearing is a significant component in rotating machinery. Suitable bearing fault detection and diagnosis (FDD) is vital to maintaining machine operations in a safe and healthy state. To address this issue, an extended observer-based FDD method is proposed, which uses a variable structure feedback linearization observer (FLO). The traditional feedback linearization observer is stable; however, this technique suffers from a lack of robustness. The proposed variable structure technique was used to improve the robustness of the fault estimation while reducing the uncertainties in the feedback linearization observer. The effectiveness of the proposed FLO procedure for the identification of outer, inner, and ball faults was tested using the Case Western University vibration dataset. The proposed model outperformed the variable structure observer (VSO), traditional feedback linearization observer (TFLO), and proportional-integral observer (PIO) by achieving average performance improvements of 5.5%, 8.5%, and 18.5%, respectively.

## 1. Introduction

The most common method to decrease the friction in rotating machinery is the use of rolling element bearings (REBs) [1]. REBs have been used in many diverse applications, such as industrial meters, aerospace, and engines [2]. Across industries, the reliability and lifespan of the rotating machine are two critical factors for its safe and continued operation. However, various parameters can reduce the bearing lifespan; such as improper installation, the presence of contaminants, and incorrect handling [3]. Thus, the design and application of stable and reliable techniques for fault detection and diagnosis (FDD) are critical for identifying various faults prior to complete machine failure.

The four main types of bearing failure are the inner, outer, ball, and cage faults [4]. When a crack or spall exists in any of these raceways, the bearing will generate impulses, depending on its dynamics. To analyze the bearing condition, different condition monitoring techniques based on acoustic emissions, stator current, shaft voltage, bearing circuit analysis, vibration, and bearing current have been studied [5]. Among these, the vibration and acoustic emission measurement techniques have been the most widely used [5,6,7,8,9,10]. Various signature analysis methods of vibration measurements have been explored to improve the performance and reliability of the condition monitoring techniques [6,7,8]. Moreover, fault detection and diagnosis can be divided into four major categories: (a) Signal-based [7,11,12,13,14,15], knowledge-based [16,17,18,19,20,21], model-based [22,23,24], and hybrid-based fault diagnosis [12,25,26]. To improve these methods, wavelet analysis has been introduced [15]. The drawback of this technique is that it reduces the frequency resolution at high frequency and the time resolution at low frequency. The main challenge of signal-based FDD is the reliability of the diagnosis in the presence of uncertainties and external disturbances [1,27]. To address this issue, statistical features extracted from the signals and machine learning algorithms, such as support vector machine (SVM) and proximal support vector machine [16], have been used in the literature. Recently, several deep learning techniques such as deep autoencoders [17], artificial neural networks (ANNs), and hierarchical convolution networks have been introduced by various researchers for signal-based FDD [18,19,20,21]. Meanwhile, the diagnosis decision in the knowledge-based approach is fully dependent on the data and on proper tuning, using the various hyper-parameters [28]. The model-based method, on the other hand, is relatively simple and can be easily applied if the appropriate dynamics of the target system are available.

In this paper, therefore, a model-reference fault detection and diagnosis technique for the rolling element bearing is proposed [1,18,29,30]. Various researchers have used observational techniques, based on different algorithms. Examples include the proportional-integral (PI) technique [31,32], proportional multiple-integral (PMI) method [33,34,35], descriptor technique [36,37], adaptive methods [38,39,40], sliding mode techniques [41,42,43,44], and feedback linearization techniques [45,46]. Linear observer methods (e.g., PI and PMI) have been used in various applications for FDD, but these techniques have challenges in the presence of uncertainties [47,48]. To solve the challenge of linear observers, nonlinear observer techniques have been recommended [44,45,46]. One of the well-known nonlinear observation techniques for FDD is the sliding mode observer [41,42,43,44]. Apart from the numerous positive attributes of the sliding mode observer, such as stability and reliability, this technique has the challenge of a chattering phenomenon [47], with solutions oscillating about a local minimum. To avoid chattering, a proposed feedback linearization observer is recommended in this research. Feedback linearization is a procedure for system linearization, but it is ultimately a nonlinear control theory technique. This observer works based on the system behavior, and thus the output performance can be excellent if the system’s dynamics are adequately known. The traditional feedback linearization observer is stable; however, this technique suffers in its robustness. The variable structure technique was used to improve the robustness with respect to fault estimation and the uncertainties in the feedback linearization observer. The efficacy of the proposed feedback linearization observer (PFLO) approach was validated using data collected from Case Western University rolling element bearing tests [49]. The remainder of this paper is organized as follows. The research problem is described in Section 2. The proposed feedback linearization observer is presented in Section 3. Results and discussion are provided in Section 4. In Section 5, conclusions are presented.

## 2. Problem Statement and Diagnosis Objective

Based on references [39,42], the bearing model is presented as a five-degree-of-freedom nonlinear and time-varying system. The energy formulation for REB is defined as in the following equation [42].

(1) $$F_{\left(q\right)}=(I_{\left(q\right)}+\Delta I_{\left(q\right)})[\ddot{q}]+(C_{\left(q\right)}+\Delta C_{\left(q\right)})[\dot{q}]+(g_{\left(q\right)}+\Delta g_{\left(q\right)})[q]+N+\psi$$

The uncertainty is defined as in the following equation.

(2) $$\Delta_{d}=\Delta I_{\left(q\right)}[\ddot{q}]+\Delta C_{\left(q\right)}[\dot{q}]+\Delta g_{\left(q\right)}[q]$$

Based on Equations (1) and (2), the dynamic equation of the bearing is represented as follows:

(3) $$F_{\left(q\right)}=I_{\left(q\right)}[\ddot{q}]+C_{\left(q\right)}[\dot{q}]+g_{\left(q\right)}[q]+\Delta_{d}+N+\psi$$

To model the above system equation, we have:

(4) $$\left[\ddot{q}]={I^{-1}}_{\left(q\right)}[F_{\left(q\right)}-C_{\left(q\right)}[\dot{q}]-g_{\left(q\right)}[q]-\Delta_{d}-N-\psi \right]$$

If $\Omega =C_{\left(q\right)}[\dot{q}]+g_{\left(q\right)}[q]+N$, and $\Gamma ={I^{-1}}_{\left(q\right)}[\Delta_{d}+\psi ]$, then Equation (4) is re-written as follows:

(5) $$[\ddot{q}]={I^{-1}}_{\left(q\right)}[F_{\left(q\right)}-\Omega_{\left(q,\dot{q}\right)}]-\Gamma_{\left(q,\dot{q},t\right)}$$

In a healthy condition, the bounded uncertainty is defined as follows:

(6) $$if(\psi =0)\to \left\|{I^{-1}}_{\left(q\right)}\times \Delta_{d}\right\|\leq \sigma_{n}$$

In a faulty condition, Equation (6) can be re-written as follows:

(7) $$if(\psi \neq 0)\to \left\|{I^{-1}}_{\left(q\right)}\times (\psi +\Delta_{d})\right\|>\sigma_{n}$$

Based on the REB dynamic formulations, the system’s equation is very complicated and uncertain, and the task of designing a reliable FDD technique is, therefore, a significant challenge. To solve these challenges, this research proposes the use of a feedback linearization observer. The literature contains different types of model-reference techniques that have been introduced for fault diagnosis in different systems. Why then, does this research introduce a nonlinear technique?

To answer this question, we introduce two problems and solutions, as follows:

**Problem:** Obtaining a bearing fault diagnosis based on a linear observer.

**Solution:** The auto regressive with exogenous input (ARX)-Laguerre PI-observer is introduced to perform fault diagnosis, based on the linear technique. This technique is introduced in Section 3.1, and the results of this technique are explained in Section 4.2.

**Problem:** The accuracy of the ARX-Laguerre PI observer degrades in the presence of uncertainties and highly nonlinear conditions associated with a fault.

**Figure 1:**
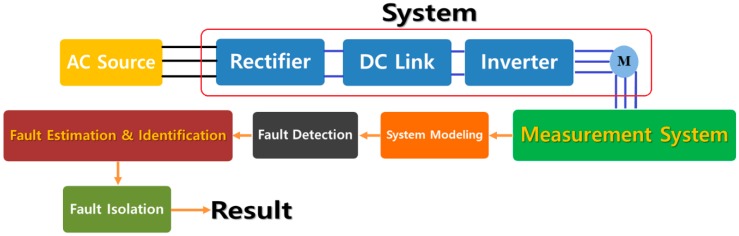
Block diagram of the system’s fault diagnosis.

**Solution:** To address this issue, a robust variable structure extended feedback linearization observer is recommended. The proposed method designs the robust fault estimator function to improve the performance of the linear ARX-Laguerre PI observer, a traditional feedback linearization observer, and a conventional variable structure observer. This proposed method is introduced in Section 3.2, and the results are described in Section 4.2. Figure 1 shows the fault diagnosis steps in this research paper.

## 3. Proposed Method

Based on references [47,50], the Case Western Reserve University (CWRU) bearing is modelled as a 5-DOF (degrees of freedom) system. Let us consider $Z_{1}=q\;\mathrm{and}\;Z_{2}=\dot{q}$. The Lagrange formulation of the bearing in Equation (3) can be written in state space form as:

(8) $$\left\{ \begin{array}{l} {\dot{Z}}_{1}=Z_{2}=\dot{q}\\ {\dot{Z}}_{2}=\ddot{q}=f(Z_{1},Z_{2},u)+\Delta_{d}(Z_{1},Z_{2},t)+\psi \\ W={\left(C\right)}^{T}Z_{1} \end{array}\right.$$

where $f(Z_{1},Z_{2},u)={I^{-1}}_{\left(q\right)}[F_{\left(q\right)}-\Omega_{\left(q,\dot{q}\right)}]$ and $\psi =\psi_{b}+\psi_{i}+\psi_{o}$. To validate the proposed method, we will compare this method with a state-of-the-art proportional-integral observation (PIO) technique [32,47], the traditional feedback linearization observer (TFLO) in Equation (9), and a variable structure observer (VSO) [47].

### 3.1. Proportional-Integral Observer (PIO)

In the first step, the proportional-integral observer (PIO) is recommended for the FDD of the bearing. This technique is linear and models the fault based on the integral term. The formulation of the PIO technique for FDD in the bearing is defined as follows [32]:

(9) $$\left\{ \begin{array}{l} \hat{Z}(k)=[\alpha \hat{Z}(k-1)+\beta_{w}\hat{W}(k-1)+\beta_{u}u(k-1)]+{\hat{\Delta }}_{d}(k-1)\\ +\hat{\psi }(k-1)+K_{p}[W(k-1)-\hat{W}(k-1)]]\\ \hat{W}(k)={\left(C_{\alpha }\right)}^{T}\hat{Z}(k) \end{array}\right.$$

Based on the ARX-Laguerre PI observer technique, the fault is modeled based on the linear integral theorem, as follows:

(10) $$\hat{\psi }(k)=\hat{\psi }(k-1)+K_{i}[W(k-1)-\hat{W}(k-1)]$$

Based on reference [32], the coefficients $\left(\alpha ,\beta_{w}\right)$ and $\beta_{u}$ are calculated as follows:

(11) $$\alpha =\left[\begin{array}{cc} \alpha_{w} & O_{N_{a},N_{b}}\\ O_{N_{b},N_{a}} & \alpha_{u} \end{array}\right]$$

The $\alpha_{w}$ and $\alpha_{u}$ coefficients can be written as [32,47]

(12) $$\left\{ \begin{array}{l} \alpha_{w}=\left[\begin{array}{cccc} \zeta_{a} & 0 & \ldots & 0\\ 1-{\zeta_{a}}^{2} & \ldots & \ldots & 0\\ -\zeta_{a}(1-{\zeta_{a}}^{2}) & \ldots & \ldots & 0\\ \begin{array}{c} \ldots \\ \ldots \\ {\left(-\zeta_{a}\right)}^{N_{a}-1}(1-{\zeta_{a}}^{2}) \end{array} & \begin{array}{c} \ldots \\ \ldots \\ \ldots \end{array} & \begin{array}{c} \ldots \\ \ldots \\ \ldots \end{array} & \begin{array}{c} 0\\ 0\\ \zeta_{a} \end{array} \end{array}\right]\\ \alpha_{u}=\left[\begin{array}{cccc} \zeta_{b} & 0 & \ldots & 0\\ 1-{\zeta_{b}}^{2} & \ldots & \ldots & 0\\ -\zeta_{b}(1-{\zeta_{b}}^{2}) & \ldots & \ldots & 0\\ \begin{array}{c} \ldots \\ \ldots \\ {\left(-\zeta_{b}\right)}^{N_{b}-1}(1-{\zeta_{b}}^{2}) \end{array} & \begin{array}{c} \ldots \\ \ldots \\ \ldots \end{array} & \begin{array}{c} \ldots \\ \ldots \\ \ldots \end{array} & \begin{array}{c} 0\\ 0\\ \zeta_{b} \end{array} \end{array}\right] \end{array}\right.$$

Based on the recursive nature of Equation (10) when inserted into Equation (9), it is clear that this technique is prone to large fluctuations in uncertain and highly nonlinear conditions. To address this issue, a nonlinear model-reference fault estimation algorithm is recommended.

### 3.2. Variable Structure Extended Feedback Linearization Observer (FLO)

**Figure 2:**
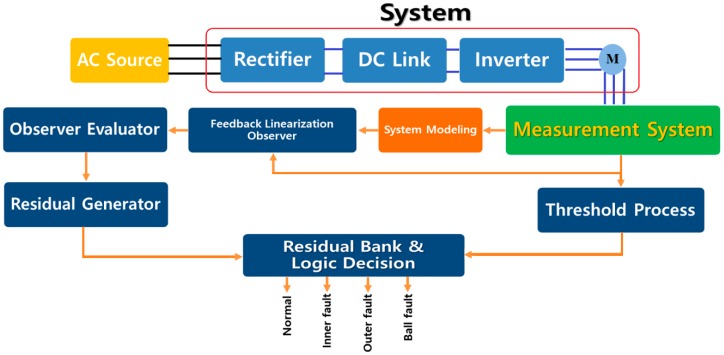
Extended variable structure feedback linearization observer for fault diagnosis in bearings.

The proposed methodology comprises five major parts: (a) feedback linearization observer, (b) observer evaluator, (c) residual generator, (d) threshold process, and (e) residual bank and logic decision. The traditional feedback linearization observer (TFLO) offers a nonlinear approach to find an optimized estimation of the system and fault. This technique is stable, but it has some issues in the presence of uncertainties. To evaluate the feedback linearization observer, a variable structure observer is recommended. This technique is one of the highly robust fault detectors for uncertain and faulty systems. Figure 2 illustrates the overall proposed mechanism for bearing fault diagnosis. The TFLO model adaptively improves the linearized model. The state space traditional feedback linearization observer is defined in the following formulation:

(13) $$\left\{ \begin{array}{l} {\dot{\hat{Z}}}_{1}={\hat{Z}}_{2}+K_{p_{1}}e_{1},(e_{1}=Z_{1}-{\hat{Z}}_{1})\\ {\dot{\hat{Z}}}_{2}=f(Z_{1},{\hat{Z}}_{2},u)+{\hat{I}}^{-1}(K_{p_{2}}e_{2})+\hat{\psi }(k-1),(e_{2}={\dot{\hat{Z}}}_{1}-{\hat{Z}}_{2})\\ \hat{W}={\left(C_{\alpha }\right)}^{T}{\hat{Z}}_{1} \end{array}\right.$$

The fault is modelled based on the following definition:

(14) $$\hat{\psi }(k)={\hat{I}}^{-1}(\hat{\psi }(k-1)+K_{i_{1}}(W(k-1)-\hat{W}(k-1)))$$

The traditional feedback linearization observer is stable; however, this technique suffers from a lack of robustness. A variable structure observer (VSO) is one of the nonlinear and robust fault detectors for uncertain and faulty systems. The state space formulation for the VSO is defined follows [47]:

(15) $$\left\{ \begin{array}{l} \begin{array}{cc} {\dot{\hat{Z}}}_{1}={\hat{Z}}_{2}+\gamma_{a}.\mathrm{sgn}(e_{1}), & \left(e_{1}=Z_{1}-{\hat{Z}}_{1}\right) \end{array}\\ \begin{array}{cc} {\dot{\hat{Z}}}_{2}=f(Z_{1},{\hat{Z}}_{2},u)+\gamma_{b}.\mathrm{sgn}(e_{2}), & \left(e_{2}={\dot{\hat{Z}}}_{1}-{\hat{Z}}_{2}\right) \end{array}\\ \hat{W}={\left(C_{\alpha }\right)}^{T}{\hat{Z}}_{1} \end{array}\right.$$

According to reference [47], the VSO suffers from a chattering phenomenon. To address the issues of the traditional variable structure observer and feedback linearization observer, the robust variable structure technique was applied to the feedback linearization observer. The fault estimate based on the variable structure technique is defined as follows:

(16) $$\left\{ \begin{array}{l} K_{z}{\left\|W(k-1)-\hat{W}(k-1)\right\|}^{0.5}\mathrm{sgn}(W(k-1)-\hat{W}(k-1))-\hat{\chi }={\hat{\Delta }}_{d}(Z_{1},Z_{2},t)+\hat{\psi }\\ \hat{\dot{\chi }}=-K_{z_{0}}\times \mathrm{sgn}(W(k-1)-\hat{W}(k-1)) \end{array}\right.$$

Based on Equations (15) and (16), the proposed variable structure extended feedback linearization observer is defined as follows:

(17) $$\left\{ \begin{array}{l} {\dot{\hat{Z}}}_{1}={\hat{Z}}_{2}+K_{p_{1}}e_{1},(e_{1}=Z_{1}-{\hat{Z}}_{1})\\ {\dot{\hat{Z}}}_{2}=f(Z_{1},{\hat{Z}}_{2},u)+{\hat{I}}^{-1}(K_{p_{2}}e_{2})+{\hat{\Delta }}_{d}(Z_{1},Z_{2},t)+\hat{\psi },(e_{2}={\dot{\hat{Z}}}_{1}-{\hat{Z}}_{2})\\ \hat{W}={\left(C_{\alpha }\right)}^{T}{\hat{Z}}_{1} \end{array}\right.$$

The fault estimation formulation is defined as follows:

(18) $$\left\{ \begin{array}{l} \hat{\psi }(k)=\hat{\psi }(k-1)+K_{i_{1}}(W(k-1)-\hat{W}(k-1))+K_{z}{\left\|W(k-1)-\hat{W}(k-1)\right\|}^{0.5}\mathrm{sgn}(W(k-1)-\hat{W}(k-1))-\hat{\chi }\\ \hat{\dot{\chi }}=-K_{z_{0}}\times \mathrm{sgn}(W(k-1)-\hat{W}(k-1)) \end{array}\right.$$

Based on Equations (8) and (17), the residual signal is defined as follows:

(19) $$r=W(k)-\hat{W}(k)$$

where $W(k)\;\mathrm{and}\;\hat{W}(k)$ are calculated in Equations (8) and (17), respectively. After obtaining the stability, the state space estimation of ${\hat{Z}}_{1},{\hat{Z}}_{2}$ converges to $Z_{1},Z_{2}$, and the estimation error converges to zero and $K_{z}{\left\|W(k-1)-\hat{W}(k-1)\right\|}^{0.5}\mathrm{sgn}(W(k-1)-\hat{W}(k-1))=0$. More specifically, the convergence conditions are specified by the following criteria:

(20) $$\begin{array}{l} {\hat{\Delta }}_{d}(Z_{1},Z_{2},t)+\hat{\psi }-K_{z}{\left\|W(k-1)-\hat{W}(k-1)\right\|}^{0.5}\mathrm{sgn}(W(k-1)-\hat{W}(k-1))-\hat{\chi }=0\to \\ K_{z}{\left\|W(k-1)-\hat{W}(k-1)\right\|}^{0.5}\mathrm{sgn}(W(k-1)-\hat{W}(k-1))=0\to \\ {\hat{\Delta }}_{d}(Z_{1},Z_{2},t)+\hat{\psi }>\sigma_{n} \end{array}$$

When the variable structure observer is applied to the feedback linearization observer, as in Equation (18), the challenge of uncertainties and fault estimation can be solved in finite time. If the states of the system are bounded as $\left|f(Z_{1},{\hat{Z}}_{2},u)\right|<J^{+}$, to guarantee the stability and convergence, the variable structure fault estimator gains $K_{z_{0}}\;\mathrm{and}\;K_{z}$ is calculated as follows:

(21) $$\{\begin{array}{l} K_{z_{0}}=1.1J^{+}\\ K_{z}=1.5\sqrt{J^{+}} \end{array}$$

Based on the Lyapunov theorem, the Lyapunov function of the proposed observer is defined by the following equation.

(22) $$V(x)=2K\left|W(k-1)-\hat{W}(k-1)\right|+\frac{1}{2}{\hat{\chi }}^{2}+\frac{1}{2}{\left(K_{z}{\left|W(k-1)-\hat{W}(k-1)\right|}^{0.5}\mathrm{sgn}(W(k-1)-\hat{W}(k-1))-\hat{\chi }\right)}^{2}$$

Based on Equation (22), the Lyapunov derivative function is proposed in Equation (23).

(23) $$\begin{array}{l} \dot{V}(x)=\frac{1}{{\left|W(k-1)-\hat{W}(k-1)\right|}^{0.5}}\left[\begin{array}{cc} W(k-1)-\hat{W}{\left(k-1\right)}^{0.5}\mathrm{sgn}(W(k-1)-\hat{W}(k-1)) & \hat{\chi } \end{array}\right]\\ \frac{K_{z}}{2}\left[\begin{array}{cc} {K_{z}}^{2} & -K_{z}\\ -K_{z} & 1 \end{array}\right]\left[\begin{array}{c} \left(W(k-1)-\hat{W}{\left(k-1\right)}^{0.5}\mathrm{sgn}((W(k-1)-\hat{W}(k-1)\right)\\ \hat{\chi } \end{array}\right]\\ +\frac{\Delta_{d}(Z_{1},Z_{2},t)-{\hat{\Delta }}_{d}(Z_{1},Z_{2},t)}{{\left|\left(W(k-1)-\hat{W}(k-1\right)\right|}^{0.5}}\left[\begin{array}{cc} \frac{{K_{z}}^{2}}{2} & \frac{-K_{z}}{2} \end{array}\right]\left[\begin{array}{c} \left(W(k-1)-\hat{W}{\left(k-1\right)}^{0.5}\mathrm{sgn}((W(k-1)-\hat{W}(k-1)\right)\\ \hat{\chi } \end{array}\right] \end{array}$$

The uncertainty estimation accuracy band is defined by the following

(24) $$\left|\Delta_{d}(X_{1},X_{2},t)-{\hat{\Delta }}_{d}(X_{1},X_{2},t)\right|\leq \varphi {\left|W(k-1)-\hat{W}(k-1)\right|}^{0.5}$$

Based on Equation (24) the Lyapunov derivative function is applied to Equation (24) and rewritten in Equation (25).

(25) $$\begin{array}{l} \dot{V}(x)\leq \frac{-1}{{\left|W(k-1)-\hat{W}(k-1)\right|}^{0.5}}\left[\begin{array}{cc} {\left(W(k-1)-\hat{W}(k-1)\right)}^{0.5}\mathrm{sgn}(W(k-1)-\hat{W}(k-1)) & \hat{\chi } \end{array}\right]\frac{K_{Z}}{2}\\ \left[\begin{array}{cc} {K_{Z}}^{2}-(K_{Z})\Delta_{d}(X_{1},X_{2},t)-{\hat{\Delta }}_{d}(X_{1},X_{2},t) & -K_{Z}\\ -(K_{Z}+2(\Delta_{d}(X_{1},X_{2},t)-{\hat{\Delta }}_{d}(X_{1},X_{2},t))) & 1 \end{array}\right]\left[\begin{array}{c} W(k-1)-\hat{W}{\left(k-1\right)}^{0.5}\mathrm{sgn}(W(k-1)-\hat{W}(k-1))\\ \hat{\chi } \end{array}\right] \end{array}$$

Based on reference [51], if $\frac{K_{Z}}{2}\left[\begin{array}{cc} {K_{Z}}^{2}-(K_{Z})\Delta_{d}(X_{1},X_{2},t)-{\hat{\Delta }}_{d}(X_{1},X_{2},t) & -K_{Z}\\ -(K_{Z}+2(\Delta_{d}(X_{1},X_{2},t)-{\hat{\Delta }}_{d}(X_{1},X_{2},t))) & 1 \end{array}\right]>0$ thus, $\dot{V}<0$. Thus, it can converge to zero in finite time. Based on Equation (20) and following the formulation until the detection of a fault, $\sigma_{n}$ is introduced as a normal threshold value.

(26) $$\begin{array}{l} if(\hat{\psi }=0)\to {\hat{\Delta }}_{d}(Z_{1},Z_{2},t)\leq \sigma_{n}\\ if(\hat{\psi }\neq 0)\to {\hat{\Delta }}_{d}(Z_{1},Z_{2},t)+\hat{\psi }>\sigma_{n} \end{array}$$

The threshold values for various types of faults have been calculated by different techniques. In this research, the variable structure technique is recommended, as follows [47]:

(27) $$\sigma_{\left(n,b,i\right)}=I({\hat{Z}}_{1}).K_{z_{\alpha }}\mathrm{sgn}(\lambda_{z}e+{\left(\frac{\lambda_{z}}{2}\right)}^{2}\sum e)$$

where $e=(W(k-1)-\hat{W}(k-1))$. The following respective formulations are used for the ball, inner, and outer fault identification.

(28) $$\begin{array}{l} if:\hat{\psi }<\sigma_{n},\hat{\psi }<\sigma_{b},\hat{\psi }<\sigma_{i}\to \hat{\psi }=0\\ if:\hat{\psi }>\sigma_{n},\hat{\psi }<\sigma_{b},\hat{\psi }<\sigma_{i}\to \hat{\psi }={\hat{\psi }}_{b}\\ if:\hat{\psi }>\sigma_{n},\hat{\psi }>\sigma_{b},\hat{\psi }<\sigma_{i}\to \hat{\psi }={\hat{\psi }}_{i}\\ if:\hat{\psi }>\sigma_{n},\hat{\psi }>\sigma_{b},\hat{\psi }>\sigma_{i}\to \hat{\psi }={\hat{\psi }}_{o} \end{array}$$

**Figure 3:**
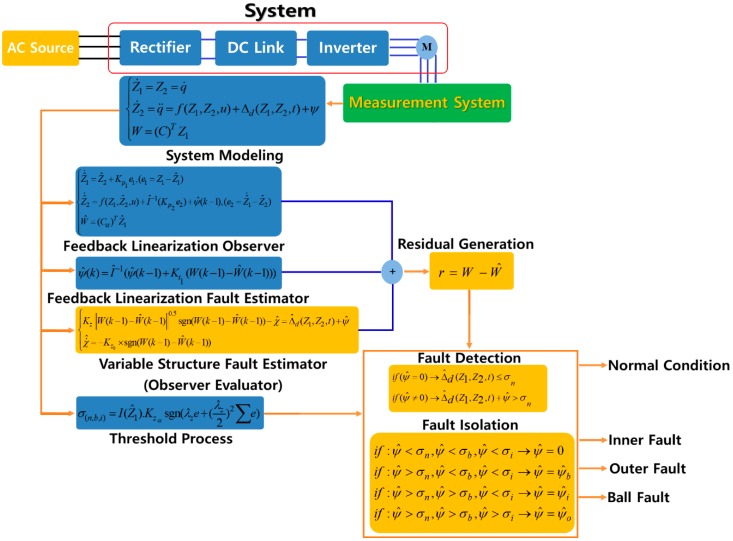
The proposed extended variable structure of the feedback linearization observer method of fault diagnosis.

According to Equation (28), the ball, inner, and outer faults are isolated whenever the residuals $\left({\hat{\psi }}_{b},{\hat{\psi }}_{i},{\hat{\psi }}_{o}\right)$ overshoot their corresponding thresholds $\left(\sigma_{n},\sigma_{b},\sigma_{i}\right)$, respectively. Figure 3 shows the block diagram of the proposed feedback linearization observer. As presented in Figure 3, the fault estimation and identification process were designed to estimate each faulty state (e.g., normal, inner (IR), outer (OR), and ball in this study). In this design, the defective signal is highly sensitive to one of the residual signals, and it is robust to the other faults and disturbances. The outline realization of the extended variable structure feedback linearization observer method for the fault diagnosis of the bearing is summarized in Algorithm 1.

**Algorithm 1** Extended variable structure feedback linearization observer for fault diagnosis of the bearing1: Bearing vibration modelling (8) 2: Run the feedback linearization observer (13), (14) 3: Run the observer evaluator based on the VSO method (16), (18) 4: Run the residual generator (19) 5: Run the threshold value based on variable structure technique (27) 6: Run the decision logic and residual bank for fault detection and diagnosis (28)

## 4. Results and Analysis

According to reference [47], the vibration bearing behavior was modeled using a 5-DOF mathematical technique, and the parameters for this modeling are given in Table 1.

### 4.1. Bearing Data

**Figure 4:**
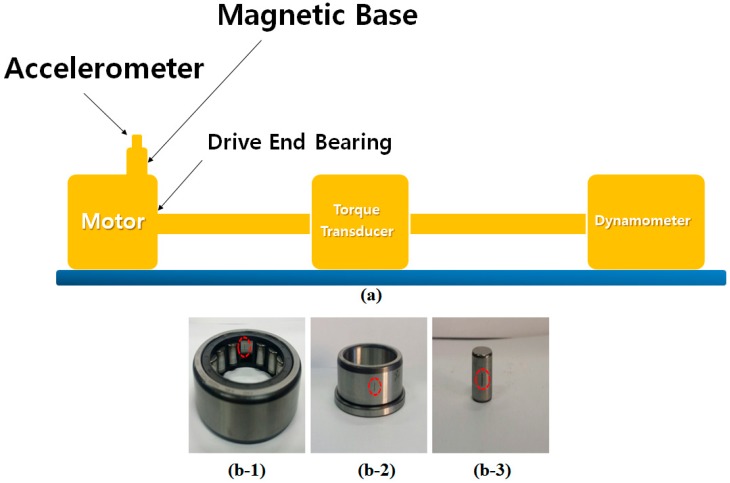
An overview of the experimental setup and bearing faults: (**a**) schematic, (**b-1**) inner fault, (**b-2**) outer fault, and (**b-3**) ball fault.

The vibration data were collected from a 6205-2RS JEM SKF roller bearing installed in a rotary motor. Based on Figure 4, a 2-horsepower (hp), three phase induction motor was connected to a torque transducer and a dynamometer to apply different loads, ranging from 0 hp to 3 hp [2]. The vibration sensor (accelerometers) was attached to the roller bearing for data collection. The vibration signals were collected for normal and faulty conditions with a 12 kHz sampling rate. The rotation velocities of the induction motor also varied from 1730 rpm to 1790 rpm. Table 2 presents the details of the Case Western University bearing dataset. Based on the work in reference [49], which is outlined in Table 2, three different crack sizes, four different motor loads, and four different motor speeds were seeded at different positions of the bearing.

### 4.2. Performance Measurement

We compared the proposed variable structure feedback linearization observer proportional integral observation (PIO) technique [32,47], traditional feedback linearization observer (TFLO), and variable structure observer (VSO) [47] for performance analysis. Based on Equations (27) and (28), to define the threshold of each type of fault, we calculated the residue of the four different states.

(29) $$-\sigma_{n}<\hat{\psi }<\sigma_{n}\to -1<\hat{\psi }<+1$$

(30) $$\{\begin{array}{c} -\sigma_{b}<{\hat{\psi }}_{b}<-\sigma_{n}\\ \sigma_{n}<{\hat{\psi }}_{b}<\sigma_{b} \end{array}\to \left\{ \begin{array}{l} 1<{\hat{\psi }}_{b}<2\\ -2<{\hat{\psi }}_{b}<-1 \end{array}\right.$$

(31) $$\{\begin{array}{c} -\sigma_{i}<{\hat{\psi }}_{i}<-\sigma_{b}\\ \sigma_{b}<{\hat{\psi }}_{i}<\sigma_{i} \end{array}\to \left\{ \begin{array}{l} 2<{\hat{\psi }}_{i}<4\\ -4<{\hat{\psi }}_{b}<-2 \end{array}\right.$$

(32) $$\{\begin{array}{c} -\sigma_{i}>{\hat{\psi }}_{o}\\ {\hat{\psi }}_{o}>\sigma_{i} \end{array}\to \left\{ \begin{array}{l} {\hat{\psi }}_{o}>4\\ {\hat{\psi }}_{o}<-4 \end{array}\right.$$

**Figure 5:**
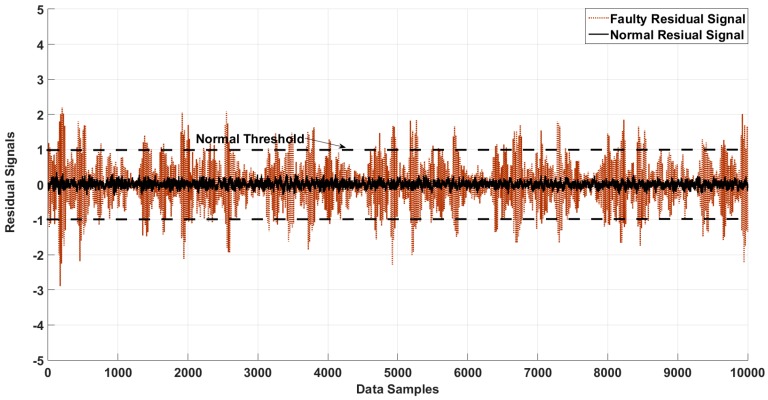
Residual of the normal and faulty states of the bearing and the normal threshold for fault detection.

Three different severity levels (0.007, 0.021, and 0.021 inches) were employed in this study. Figure 5 shows the normal threshold values, and residual signals for normal and faulty signals from the forth dataset in the Case Western University fault detection experiments, based on the proposed method.

**Figure 6:**
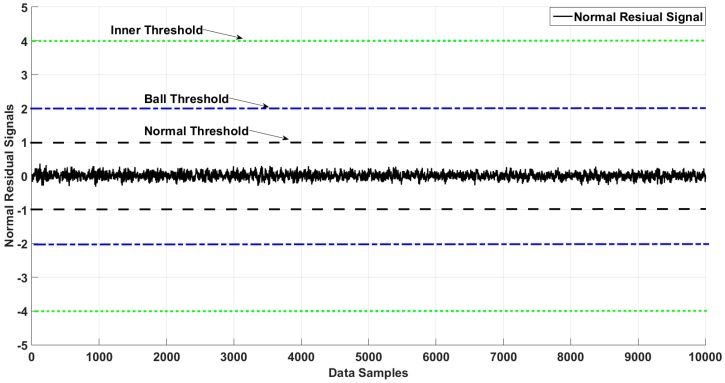
Residual of the normal state of the bearing and its thresholds.

**Figure 7:**
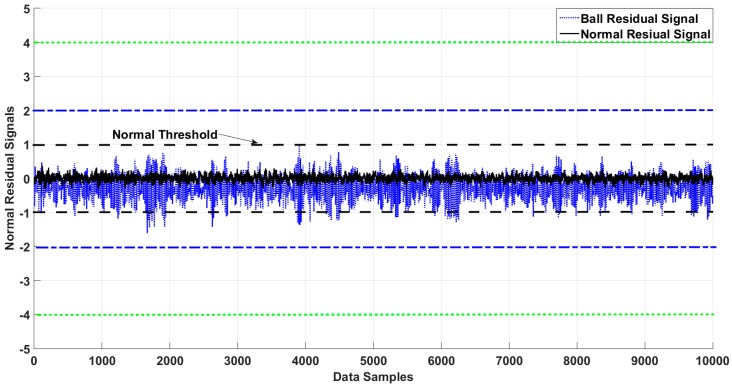
Residual of the ball and normal states of the bearing and their thresholds.

**Figure 8:**
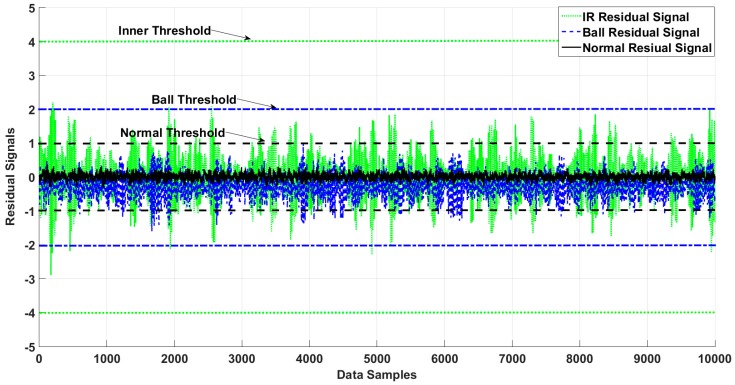
Residual of the inner, ball, and normal states of the bearing and their thresholds.

**Figure 9:**
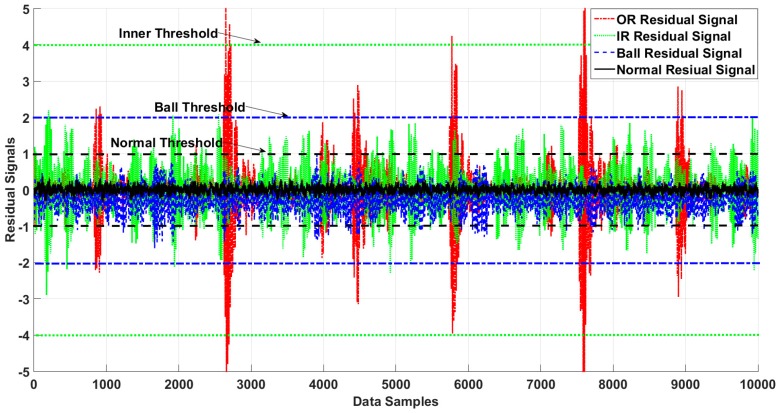
Residual of the outer, inner, ball, and normal states of the bearing and their thresholds.

Figure 6, Figure 7, Figure 8 and Figure 9 illustrate the residual signals and threshold values of the normal and faulty conditions for fault diagnosis, based on the proposed method. Figure 6 shows the threshold values and residual of the normal bearing signal from the third dataset of the Case Western University experiments. Based on Equations (26) and (28), the range of the normal threshold value was between [−1, 1]. The normal and ball residual signals and threshold values are illustrated in Figure 7. Figure 8 shows the normal, ball, and inner residual signals and the threshold values for identifying the faults. The residual signal of the outer fault is illustrated in Figure 9.

**Figure 10:**
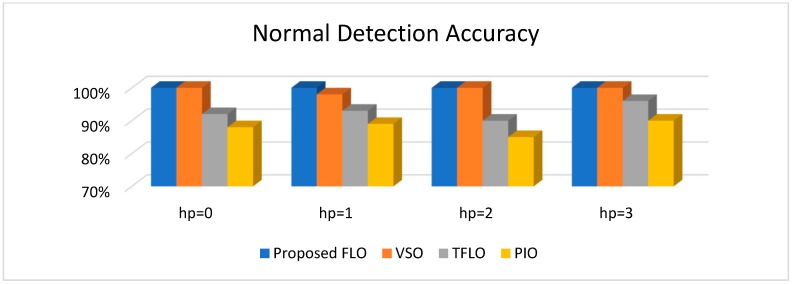
Normal detection accuracy for proposed feedback linearization observer (FLO), variable structure observer (VSO), traditional feedback linearization observer (TFLO), and proportional-integral observer (PIO).

**Figure 11:**
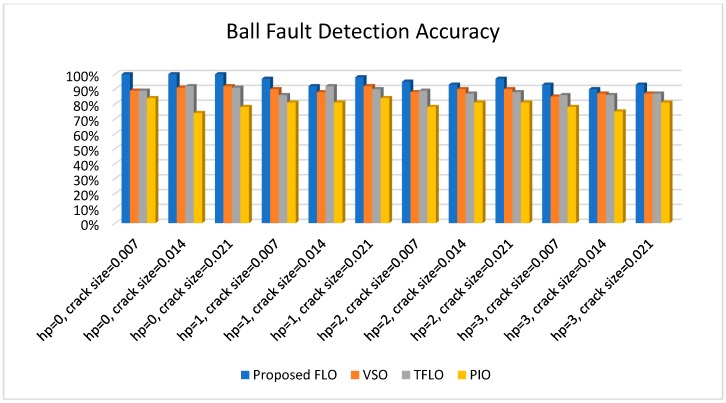
Ball fault detection accuracy for proposed FLO, VSO, TFLO, and PIO models.

**Figure 12:**
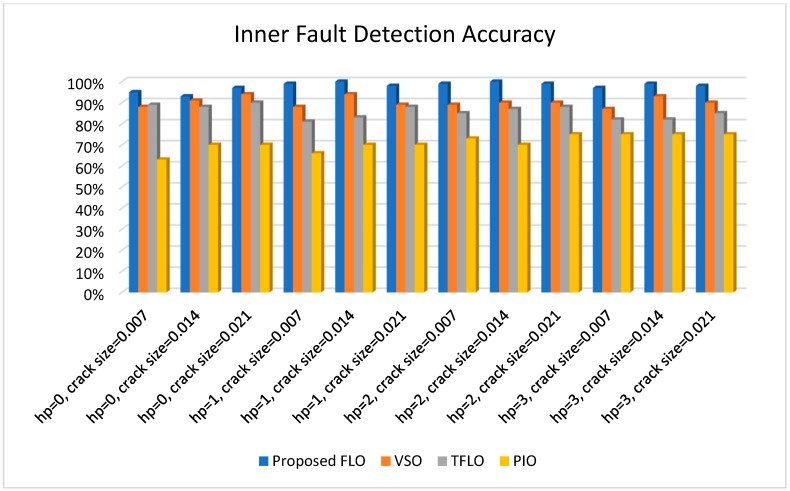
Inner fault detection accuracy for proposed FLO, VSO, TFLO, and PIO models.

**Figure 13:**
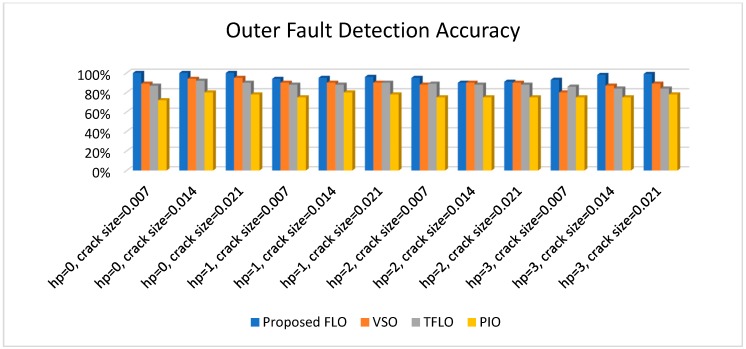
Inner fault detection accuracy for proposed FLO, VSO, TFLO, and PIO models.

Figure 10, Figure 11, Figure 12 and Figure 13 illustrate the detection and diagnostic accuracy of proposed FLO, TFLO, VSO and PIO techniques for the normal condition, faulty ball condition, inner defect states, and outer failure, respectively. The diagnostic accuracy is reported as a percentage of the rate of correct detection in all data.

In Figure 10, Figure 11, Figure 12 and Figure 13, we can see that the average rates of defect detection are 97.5% for the proposed FLO, 92% for the VSO technique, 89% for the TFLO method, and 79% for the PIO method. Consequently, the average performance improvements for the proposed procedure were 5.5%, 8.5%, and 18.5% in comparison to the VSO, TFLO, and PIO models, respectively.

Table 3, Table 4 and Table 5 present the diagnostic accuracy for variant motor speed (e.g., 1730 rpm, 1750 rpm, 1772 rpm, and 1790 rpm) in different size of cracks (e.g., 0.007 in, 0.014 in, and 0.021 in) and conditions (normal, ball faulty, inner faulty, and outer defect) of the proposed FLO, TFLO, SMO, and PIO.

## 5. Conclusions

As a result of the uncertain and nonlinear parameters of REB dynamics, including noisy vibration signals, the task of accurate fault diagnosis in the bearing system is a formidable challenge. To better deal with nonlinearities in the residual signal, this paper introduced a model-based technique for fault diagnosis in bearings using a variable structure feedback linearization observer. The generation of a robust residual signal based on the variable structure feedback linearization observer was the first step in the detection and identification of a faulty REB. The power of the proposed technique to diagnose REB faults was demonstrated using the Case Western University vibration dataset. As a result, the average performance improvements for the recommended procedure were 5.5%, 8.5%, and 18.5%, compared with the VSO, TFLO, and PIO models, respectively. In the future, a robust hybrid technique based on the feedback linearization observation method will be designed to enhance the performance of fault diagnosis. The hybrid extended state observer will be combined with a system estimation technique, and an intelligent extended state robust feedback linearization observer to improve the performance of fault diagnosis in a faulty system.

## Figures and Tables

**Table 1:** Rolling element bearing (REB) modelling information [39].

Parameters	Value
Number of balls	9
Stiffness of ball	5.96×107(Nm)
Mass of outer (Kg)	2.7(Kg)
Stiffness of outer	1.31×105(Nm)
Mass of shaft (Kg)	1.36(Kg)
Stiffness of Shaft	23.3×106(Nm)
Damping	654(NSm)
Ball diameter	7.940(mm)
Pitch diameter	39.04(mm)
Defect size	7(mm)
Defect depth	2(mm)

**Table 2:** Case Western University dataset.

Dataset f = 12 kHz	Fault Types	Motor Load (hp)	Motor Speed (rpm)	Fault Crack Size (in)	Number of Samples
Dataset 1	Normal states	0	1797	0.007, 0.014, and 0.021	1395
	IR fault states	0	1797		
	OR fault states	0	1797		
	Ball fault states	0	1797		
Dataset 2	Normal states	1	1772	0.007, 0.014, and 0.021	1395
	IR fault states	1	1772		
	OR fault states	1	1772		
	Ball fault states	1	1772		
Dataset 3	Normal states	2	1750	0.007, 0.014, and 0.021	1395
	IR fault states	2	1750		
	OR fault states	2	1750		
	Ball fault states	2	1750		
Dataset 4	Normal states	3	1730	0.007, 0.014, and 0.021	1395
	IR fault states	3	1730		
	OR fault states	3	1730		
	Ball fault states	3	1730		

**Table 3:** Fault diagnosis result for variant motor speed for proposed FLO, PIO, TFLO, AND VSO for crack size = 0.007 (inch).

Algorithms		Proposed FLO				PIO				TFLO				VSO		
**Motor Speed (rpm)**	1730	1750	1772	1797	1730	1750	1772	1797	1730	1750	1772	1797	1730	1750	1772	1797
**Normal state**	100%	100%	100%	100%	90%	85%	89%	88%	96%	90%	93%	92%	100%	100%	98%	100%
**IR fault**	97%	99%	99%	99%	75%	73%	66%	63%	82%	85%	81%	89%	87%	89%	88%	88%
**OR fault**	93%	95%	94%	100%	75%	75%	75%	87%	86%	89%	88%	87%	80%	88%	90%	89%
**Ball fault**	95%	95%	97%	100%	78%	78%	81%	84%	86%	89%	86%	89%	85%	88%	90%	89%
**Average**	96.2%	97.25%	97.5%	99.7%	79.5%	77.7%	77.8%	80.5%	87.5%	88.2%	87%	89.2%	88%	91.25%	91.5%	91.5%

**Table 4:** Fault diagnosis result for variant motor speed for proposed FLO, PIO, TFLO, and VSO for crack size = 0.014 (inch).

Algorithms		Proposed FLO				PIO				TFLO				VSO		
**Motor Speed (rpm)**	1730	1750	1772	1797	1730	1750	1772	1797	1730	1750	1772	1797	1730	1750	1772	1797
**Normal state**	100%	100%	100%	100%	90%	85%	89%	88%	96%	90%	93%	92%	100%	100%	98%	100%
**IR fault**	99%	100%	100%	96%	75%	70%	70%	88%	82%	87%	83%	88%	93%	90%	94%	91%
**OR fault**	98%	94%	95%	100%	75%	75%	80%	80%	84%	88%	88%	92%	87%	90%	90%	94%
**Ball fault**	94%	94%	95%	100%	75%	81%	81%	74%	86%	87%	92%	92%	87%	90%	88%	91%
**Average**	97.7%	97%	97.5%	99%	78.7%	77.7%	80%	82.5%	86%	88%	89%	91%	91.7%	92.5%	92.5%	94%

**Table 5:** Fault diagnosis result for variant motor speed for proposed FLO, PIO, TFLO, and VSO for crack size = 0.021 (inch).

Algorithms		Proposed FLO				PIO				TFLO				VSO		
**Motor Speed (rpm)**	1730	1750	1772	1797	1730	1750	1772	1797	1730	1750	1772	1797	1730	1750	1772	1797
**Normal state**	100%	100%	100%	100%	90%	85%	89%	88%	96%	90%	93%	92%	100%	100%	98%	100%
**IR fault**	98%	99%	98%	97%	75%	75%	70%	70%	85%	88%	88%	90%	90%	90%	89%	94%
**OR fault**	99%	96%	97%	100%	78%	75%	78%	78%	84%	88%	90%	90%	89%	90%	90%	95%
**Ball fault**	94%	97%	98%	100%	81%	81%	84%	78%	87%	88%	90%	91%	87%	90%	92%	92%
**Average**	97.7%	98%	98.2%	99.2%	81%	79%	80.2%	78.5%	88%	88.5%	90.2%	90.7%	91.5%	92.5%	92.2%	95.2%

## Nomenclature

$F_{\left(q\right)}$	Force vector	$I_{\left(q\right)}$	Mass matrix
$C_{\left(q\right)}$	Stiffness matrix	$g_{\left(q\right)}$	Damping matrix
N	Nonlinear bearing parameter vector	ψ	Various types of fault
$\Delta_{d}$	Uncertain and unknown parameter	$\Delta I_{\left(q\right)}$	Unknown modeling for mass matrix
$\Delta C_{\left(q\right)}$	Unknown modeling for stiffness matrix	$\Delta g_{\left(q\right)}$	Unknown modeling for damping matrix
$\sigma_{n}$	Normal threshold value	$u=F_{\left(q\right)}$	System’s input
$\left({\dot{Z}}_{1},{\dot{Z}}_{2}\right)$	State of REB	C	Output coefficient
$\psi_{b}$	Ball fault	$\psi_{i}$	Inner fault
$\psi_{o}$	Outer fault	$\Delta_{d}(Z_{1},Z_{2},t)$	System’s uncertainty and unknown parameter
W	System’s output	$\hat{Z}(k)$	System state estimation
$\hat{W}(k)$	Measured output estimation	u(k)	Control input
${\hat{\Delta }}_{d}(k)$	Uncertainty estimation and disturbance	$\hat{\psi }(k)$	Faults estimation
$\left(C_{\alpha },K_{p},K_{i}\right)$	output, proportional, and integral coefficients, respectively	$\zeta_{a},\zeta_{b}$	Laguerre poles
$\left(O_{N_{a},N_{b}}\&O_{N_{b},N_{a}}\right)$	Null matrices	$\left(C_{\alpha },\gamma_{a},\gamma_{b}),(K_{p_{1}},K_{p_{2}},C_{\alpha }\right)$	States and output coefficients
$\left(K_{z},K_{z_{0}}),(\lambda_{z},K_{z_{\alpha }}\right)$	Coefficients	$\dot{\chi }$	Variable structure variable
$\sigma_{\left(n,b,i\right)}$	Threshold value for normal, ball, and inner	$\left(\sigma_{n},\sigma_{b},\sigma_{i}\right)$	(normal, ball, and inner) threshold values
$\left({\hat{\psi }}_{b},{\hat{\psi }}_{i},{\hat{\psi }}_{o}\right)$	(ball, inner, and outer) failures	φ,$J^{+}$	Positive constants
